# Factor Structure and Measurement Invariance of the Brief Symptom Inventory-18 Among Chinese Adults

**DOI:** 10.3389/fpsyg.2022.882815

**Published:** 2022-06-22

**Authors:** Yangwen Geng, Xiaoli Ni, Yuping Wang, Jinming Fan, Yuyan Qian, Xiaoran Li

**Affiliations:** School of Humanities and Social Sciences, Xi’an Jiaotong University, Xi’an, China

**Keywords:** Brief Symptom Inventory-18, factor structure, measurement invariance, Chinese adults, bi-factor model

## Abstract

This study aimed to investigate the factor structure and measurement invariance of the Brief Symptom Inventory-18 (BSI-18) among Chinese adults. A sample comprising 1,839 adults from four cities in Shaanxi province completed the BSI-18 and background information. The best-fitting factor structure model of the BSI-18 was verified by confirmatory factor analyses, and multigroup confirmatory factor analyses were performed to test the measurement invariance. The results indicated that the three-factor bi-factor model fitted the current data best (*χ*^2^ = 468.861, *df* = 117, CFI = 0.939, TLI = 0.920, RMSEA = 0.040, BIC = 47044.977). The configural, metric, scalar, and strict invariance models all had acceptable model fit indexes across genders, and the configural, metric, scalar invariance models with different living areas and educational levels were all acceptable. Overall, the three-factor bi-factor model of the BSI-18 shows a good fit for use with Chinese adults, making it a viable tool for effectively measuring the mental health of Chinese adults.

## Introduction

Mental health has become a topic of widespread concern across China, and there is increasing interest in being able to effectively identify and measure mental health efficiently and accurately. However, considering the huge population of China, it is a time-consuming and burdensome task to assess psychological status on a grand scale. Therefore, effective, accurate, and efficient mental health measurement tools are of great significance for the psychological evaluation of Chinese adults.

### Factor Structure of the Brief Symptom Inventory-18

The Brief Symptom Inventory-18 (BSI-18; Derogatis, 2001) is an 18-item self-report checklist that has been adapted from the Symptom Checklist-90-Revised (SCL-90-R; Derogatis, 1977) and the Brief Symptom Inventory (BSI; Derogatis, 1993). Although both the SCL-90-R and BSI contain wide ranges of measurement content and have good reliability and validity, they also suffer from issues such as time-consuming due to the length of the measures. The SCL-90 has 90 items, and the shortened BSI has 53. So it is not easy to complete for participants and may cause regular and same answers in non-clinical sample survey. The BSI-18, developed by Derogatis, is the briefest and most current instrument designed to measure three of the most prevalent psychiatric symptoms: (1) somatization—discomfort caused by perceived physical problems, including faintness or pains in chest; (2) depression—symptoms usually associated with depression, such as apathy, sadness, or suicidal thoughts; and (3) anxiety—feelings of fear, generalized tension, and panic.

Previous research has shown that the BSI-18 correlates highly with the BSI (*r* > 0.90), and that despite its brevity shows maximum sensitivity toward psychological distress assessment (Derogatis, 2001). However, when it comes to factor structure, existing findings are quite inconsistent. According to Derogatis, the original structure proposed three dimensions: somatization, depression, and anxiety. But the factor structures of the BSI-18 appear to differ in multinational clinical and non-clinical trials. Many studies have indicated that the three-factor model of the BSI-18 is the best factor structure model among samples such as Irish university students, Spanish cancer patients, and Chinese drug users (Wiesner et al., 2010; Wang et al., 2013; Calderon et al., 2020). However, Derogatis (2001) also carried out a four-factor structure in a nonclinical sample of 1,134 subjects. Two of these factors contained the exact items belonging to the dimensions of somatization and depression, while the other two factors contained the items of the anxiety dimension as originally proposed. The general anxiety factor integrated the three items related to generalized nervousness, while the panic factor comprised three items that evaluated panic symptoms. Despite these findings, Derogatis upheld the structural validity of the instrument, arguing that the latter two could still be considered as a single dimension of anxiety. Andreu et al. (2008) found support for the four-factor structure (i.e., somatization, depression, general anxiety, and panic) in non-clinical samples in Spain, but Recklitis et al. (2006) agreed with Derogatis, believing that support for the fourth dimension of the BSI-18 is weak and may be an over-extracted product. Meanwhile, Prelow et al. (2005) found that the three-factor model had no distinction in the Latino American population, and that the single-factor model was the best and most concise model for their sample.

In recent years, the bi-factor model has been used widely to solve the dimension selection problem in related fields of behavioral science as an effective method of multi-dimensional measurement tool modeling (Reise, 2012). The bi-factor model not only tests the overall situation but also imposes a secondary load on specific variables as different dimensions which is useful for determining their effects. A bi-factor model is verified using a general factor to reflect the commonality of multiple dimensions and several special factors to explain the differences between dimensions. Notably, the general factor in the bi-factor model represent a single source of common variance across all items measured. It can easily be interpreted as representing the psychological constructs that the instrument may be intended to measure (Reise, 2012). According to previous research, both the SCL-90 and BSI had a bi-factor model structure (Urbán et al., 2014), while the BSI-18 also offers a measure of general distress (total score). The bi-factor model, considering both general factor and special dimensions, is consistent with the factor structure of the BSI-18 designed by Derogatis. Perhaps the bi-factor model can explain why the three factors of the BSI (i.e., somatization, depression, and anxiety) demonstrate high correlations and comorbidities with many diseases. It has been proven that the three-factor bi-factor model of the BSI-18 is applicable to insurance practitioners in China (Li et al., 2018). However, the factor structure of the BSI-18 in other Chinese populations, especially large populations of adults with a wide variety of backgrounds, is not yet clear. Therefore, the first aim of this study was to testify the factor structure of the BSI-18 in Chinese adults.

### Significance of Measurement Invariance

Measurement invariance is an important indicator for assessing the quality of a measurement. The BSI-18 is still a relatively new instrument in China, and data on its validity and reliability in various Chinese populations are still limited. It is unclear whether the BSI-18 is equally applicable to every group in China. As a result, the second objective of this study was to determine whether the BSI-18 has the same psychometric properties across different community resident demographics. This would determine whether the BSI-18 can be widely used in the Chinese community, and whether differences between various groups can be analyzed and compared.

Communities vary greatly in terms of gender, educational level, and income, which are the main factors that can influence individual psychological characteristics. In this study, gender, educational level, and living area were chosen as the most important factors to test the measurement stability of the BSI-18. As categorical variables, these three variables can be divided into two qualitatively different groups, namely, man and women, one group with higher education and the other without, one group living in rural areas and the other living in urban areas. And, individuals in these groups do differ significantly in terms of psychological characteristics.

Research has shown that women report more physical and psychological symptoms than men (Barsky et al., 2001; Afifi, 2007). Female college students have higher anxiety levels than male college students, while males have more depressive difficulties (Gao et al., 2020). Gender differences in types of psychiatric symptoms have also been reported in the adult population, with women having a higher rate of affective disorders, anxiety disorders, and non-affective mental illnesses than men, but men having a higher rate of substance use disorders and antisocial personality disorders (Kessler et al., 1994; Vicente et al., 2006).

Meanwhile, people with different levels of education also appear to experience different psychological symptoms. According to a 2017 PhD survey report published by *Science*, 45% of PhD candidates reported experiencing depression (Woolston, 2017). The same research found that people with higher education reported more depressive symptoms (Bracke et al., 2013), and that graduate students are six times more likely to suffer from depression and anxiety than the general population (Evans et al., 2018). At the same time, studies have also shown that a higher level of education can reduce the risk of depression (Cho et al., 1998; Bauldry, 2015), and a meta-analysis of 37 studies on education and depression has confirmed the linear nature of the relationship between years of education and the prevalence of major depression (Lorant et al., 2003). It is worth exploring, however, whether people with and without higher education have the same understanding of the items on measures used. That is, whether or not these different findings are caused by subjects understanding items differently.

China’s household registration system is an important tool for allocating public funds, and the population is divided into agricultural household registration (living in rural areas) and non-agricultural household registration (living in urban areas). People with different household registration types also differ in income, education, and medical treatment. Studies have shown that people from rural regions without strong social welfare systems have lower subjective well-being, physical health, and psychological well-being than urban adults (Guo et al., 2017; Shang, 2020). Adults from rural regions have been shown to exhibit more depression symptoms (Guo et al., 2017; Shang, 2020). Furthermore, there are considerable differences in the mental health of rural and urban populations in other countries and regions, with men from rural areas demonstrating higher rates of emotional and anxiety disorders than men from urban areas (Diala and Muntaner, 2003).

### Study Objectives

BSI-18 has varied factor structures in different groups, and it is unknown which factor structure is best for Chinese adults. Therefore, the first aim of this study was to investigate the factor structure of the BSI-18 in Chinese adults as well as obtain the reliability of the BSI-18. Individual psychological symptoms exhibit differently according to gender, living area, and educational level. Because of these variances, it is important to assess the measurement equivalence of a tool and make sure these differences are real and meaningful. The second aim was to assess the measurement invariance of the BSI-18’s best-fit model between gender, living area, and educational level.

## Materials and Methods

### Participants

A total of 2,217 adults were selected using stratified random sampling. According to its geography, Shaanxi province can be divided into three different natural regions: northern Shaanxi, Guanzhong (the middle of Shaanxi), and southern Shaanxi. The economic development and typical lifestyles in each of these three regions are quite different. Four cities were selected based on regions and population distribution: Yanan for northern Shaanxi, Baoji and Weinan for Guanzhong (accounting for more than half of the province’s population), and Hanzhong for southern Shaanxi. Then, according to their gross domestic product, one district and one county with different economic levels was selected from each city. Finally, a community or village was chosen from each district and county, one with a higher economic level and one with a lower one. A random sample of residents in their communities and natural villages.

After collecting subject responses, the results of the Little’s test for the items of BSI-18 were: *χ*^2^ = 3480.276, *df* = 1,414, sig < 0.001. The results indicated that the missing values were not randomly generated. Participants consistently responded with the same answers or did not take it seriously. Therefore, samples with missing items were removed. In total, 1,839 valid questionnaires were obtained, with an effective rate of 82.95%. The mean age of participants was 43.86 (*SD* = 11.82; age range = 21–87), and 60.63% of them were women. In addition, 83.71% of the participants were married, 25.12% of them were highly educated, and 61.99% of them lived in the countryside (see Table 1 for more information).

**Table 1:** Demographic characteristics of subjects (*n* = 1,839).

	Category	Frequency	Percentage
Gender	Male	724	39.37
	Female	1,115	60.63
Living area	Rural area	1,140	61.99
	Urban area	673	36.60
	Unreported	26	1.41
Nation	Han nationality	1,818	98.86
	Other	13	0.70
	Unreported	8	0.44
Age	21–30	121	6.58
	31–40	693	37.68
	41–50	512	27.84
	51–60	174	9.46
	61–70	140	7.61
	>71	56	3.05
	Unreported	143	7.78
Educational level	Unschooled	69	3.75
	Primary school	171	9.30
	Junior high school	651	35.40
	High school	471	25.61
	Junior college	266	14.46
	Undergraduate and above	196	10.66
	Unreported	15	0.82
Marital status	Unmarried	76	4.13
	Married	1,521	82.71
	Other	169	9.19
	Unreported	73	3.97

### Measures

#### Self-Report Demographic Data Questionnaire

Socio-demographic data of the subjects were collected through self-report questionnaires, which included subjects’ gender, age, living area, ethnicity, educational level, and marital status.

#### The Brief Symptom Inventory-18

The BSI-18 (Derogatis, 2001) is a self-report screening inventory designed to assess the extent of depression, anxiety, and other physical and mental conditions experienced by the respondent throughout the past week. The scale contains a total of 18 items which are rated on a five-point Likert-type scale, ranging from 0 (not at all) to 4 (extremely). Similar to the original theoretical model, the BSI-18 has three subscales, each scored by summing up the scores of the six separate items. The global severity index (GSI) of distress is the sum of all three subscales. The higher the score, the worse the respondent’s mental health. The Chinese version of the BSI-18 was used in this study, which uses the same scoring and items as the original scale (Li et al., 2018). The internal consistency estimates reported by Derogatis (2001) for the community sample of 1,134 adults were acceptable (0.74 for somatization, 0.79 for anxiety, 0.84 for depression, and 0.89 for GSI scores). In this study, the BSI-18 also showed satisfactory Cronbach’s alpha indexes of reliability for all three dimensions (0.836–0.906) and the GSI scores (0.945).

### Procedure

All surveys were completed in the communities where the participants lived. Some participant outreach was done through free mental health lectures with questionnaires sent to those interested in participating after the lecture. Another portion of participants completed a household survey. Participants received a gift after completing the questionnaires. Before starting, all participants received guidance regarding the study and the questionnaire from trained research assistants who were able to provide assistance and clarification if participants had any questions. Assistants received rigorous training before sampling began. All the questionnaire items were fixed, and assistants were asked to read the items using a certain pronunciation and intonation. To ensure all participants understood how to answer the items, several example questions were developed by the research team. For participants without Chinese reading ability, the research assistants read the questions out loud and recorded participants’ oral responses. The study was granted ethical approval before it began, and all subjects gave their written informed consent before participating.

### Data Analysis Strategy

We used SPSS version 26.0 and Mplus 7.4 to analyze the data. First, five CFA models were estimated to identify the best fit factor structure for the Chinese adult sample. This included (a) a single-factor model that integrated all items into one factor; (b) the theoretical hypothesized three-factor model (in which items 1, 4, 7, 10, 13, and 16 loaded on the somatization factor; items 2, 5, 8, 11, 14, and 17 loaded on the depression factor; and items 3, 6, 9, 12, 15, and 18 on the anxiety factor); (c) a four-factor model (items for somatization and depression factors were the same as the three-factor model; items 3, 6, and 15 loaded on a general anxiety factor; and items 9, 12, and 18 on a panic factor); (d) a three-factor bi-factor model which added a global factor to the three-factor model; and (e) a four-factor bi-factor model which added a global factor to the four-factor model. Model fits were assessed using chi-squares, root-mean-square error of approximation (RMSEA), the Tucker Lewis Index (TLI), the comparative fit index (CFI), and the BIC. Conventional guidelines indicate that an RMSEA value ≤0.08 implies an acceptable model fit and a value ≤0.05 indicates a good model fit. Meanwhile, CFI and TLI ≥ 0.90 indicate adequate model fit (Kline, 2010). The ΔBIC value of the two models was greater than 10, indicating that the model with a smaller BIC showed a better model fit (Kuha, 2004).

Second, multi-group CFAs were used to examine the measurement invariance of the BSI-18 across gender, living area, and educational level. The configural, metric, scalar, and strict invariance were all examined across the groups. Configural invariance sets no parameters across groups and tests whether the latent variables are in the same factor structure and constitutive pattern across groups, and sets up a baseline model for the next step of invariance testing. Metric invariance sets loadings across groups based on configural invariance. It measures whether each observation has the same factor loadings on the corresponding latent variables, or whether each observed variable has the same units across groups. Scalar invariance sets both the loadings and intercepts equivalence for each group to test whether different groups have the same observation points and can explain whether there is indeed a difference between groups. Strict invariance increase sets the error variance equivalence restriction. If it is verified, it means that the differences in observed score variances across groups fully reflect the differences in latent variable variances. In terms of model comparison, we used a DIFFTEST to compare the improvement in fit between nested models and adopted the CFI difference (ΔCFI) numerical model fit index to evaluate measurement invariance (Cheung and Rensvold, 2002). According to Cheung and Rensvold (2002), the equivalent model is considered to be acceptable when ΔCFI ≤ 0.010 and ΔTLI ≤ 0.010. Moreover, the reduced Bayesian information criterion (BIC) is considered as the signal of equivalence.

Coefficient omega hierarchical (ω_H_), the hierarchical omega subscales (ω_HS_) and the explained common variances (ECVs) were calculated in Mplus7.4 to evaluate the reliability of bi-factor model. ω_H_ estimates the proportion of variance in the total score, which can be attributed to a single general factor. After controlling for the variance due to the general factor, ω_HS_ reflects the reliability of a factor score (Reise et al., 2013). When the coefficient ω_H_ is greater than 0.80, total scores can be regarded as unidimensional because of the most reliable is due to a single common factor (Rodriguez et al., 2016). Meanwhile, the coefficient ω_H_ greater than 0.80 indicates that the vast majority of reliable variance imputing to a specific factor rather than a general factor (Reise et al., 2013).

## Results

### Factor Structure

Descriptive statistics, skewness, and kurtosis were included in Table 2. Table 3 shows the fit indexes of the five competing models for the factor structure of the BSI-18. As shown in Table 3, except for the single-factor model, the remaining four models demonstrated a good fit to the data (CFIs > 0.90, TLIs > 0.90). The bi-factor models provided the best fits to this sample. In bi-factor models, the general factor and special dimensions are considered. When comparing these two bi-factor models, the three-factor bi-factor model had a smaller value of S-Bχ^2^ and a bigger value of CFI and TLI. In terms of RMSEA, the value of the four-factor bi-factor model was more than 0.080, and it had a worse fit compared to the other models. Overall, the three-factor bi-factor model provided the best fit (χ2 = 468.861, *df* = 117, CFI = 0.939, TLI = 0.920, RMSEA = 0.040, BIC = 47044.977) and was selected as the baseline model for the measurement invariance test.

**Table 2:** Descriptive statistics and skewness and kurtosis (*n* = 1,839).

	M	SD	Skewness	Kurtosis
BSI1	1.32	0.619	2.292	6.223
BSI2	1.39	0.691	1.942	4.215
BSI3	1.34	0.713	2.297	5.250
BSI4	1.24	0.601	3.019	10.429
BSI5	1.31	0.706	2.733	8.082
BSI6	1.38	0.721	2.237	5.576
BSI7	1.39	0.743	2.236	5.375
BSI8	1.36	0.756	2.555	7.007
BSI9	1.21	0.616	3.659	15.029
BSI10	1.17	0.541	3.704	15.201
BSI11	1.28	0.699	3.035	10.073
BSI12	1.18	0.565	3.790	16.306
BSI13	1.27	0.636	2.774	8.242
BSI14	1.29	0.722	3.018	9.924
BSI15	1.27	0.690	3.126	10.618
BSI16	1.33	0.725	2.619	7.433
BSI17	1.12	0.504	4.941	26.717
BSI18	1.19	0.606	3.932	16.934
BSI-18 total	23.04	8.581	2.767	8.623

**Table 3:** Goodness-of-fit indices for the five tested models of the BSI-18.

Model	S-Bχ^2^	*df*	CFI	TLI	RMSEA (90% CI)	AIC	BIC	△BIC
Single-factor	800.432^*^	135	0.868	0.884	0.052(0.048,0.055)	47789.485	48087.402	
Three-factor	620.512^*^	132	0.901	0.915	0.045(0.041,0.048)	47166.492	47480.960	606.442
Four-factor	556.065^*^	129	0.912	0.925	0.042(0.039,0.046)	46935.065	47266.084	214.876
Three-factor bi-factor	**468.861** ^*^	**117**	**0.920**	**0.939**	**0.040(0.037,0.044)**	**46647.754**	**47044.977**	**221.107**
Four-factor bi-factor	1542.737	117	0.910	0.931	0.081(0.078,0.085)	46610.846	47008.069	36.908

*χ*^2^, chi-square; df, degree of freedom; CFI, comparative fit index; TLI, Tucker-Lewis index; SRMR, standardized root-mean-square residual; RMSEA, root-mean-square error of approximation; AIC, Akaike information criterion; BIC, Bayesian information criterion; and △*BIC, change in Bayesian information criterion relative to the preceding model.* *p < 0.05. The best fitting model was in bold.

**Figure 1:**
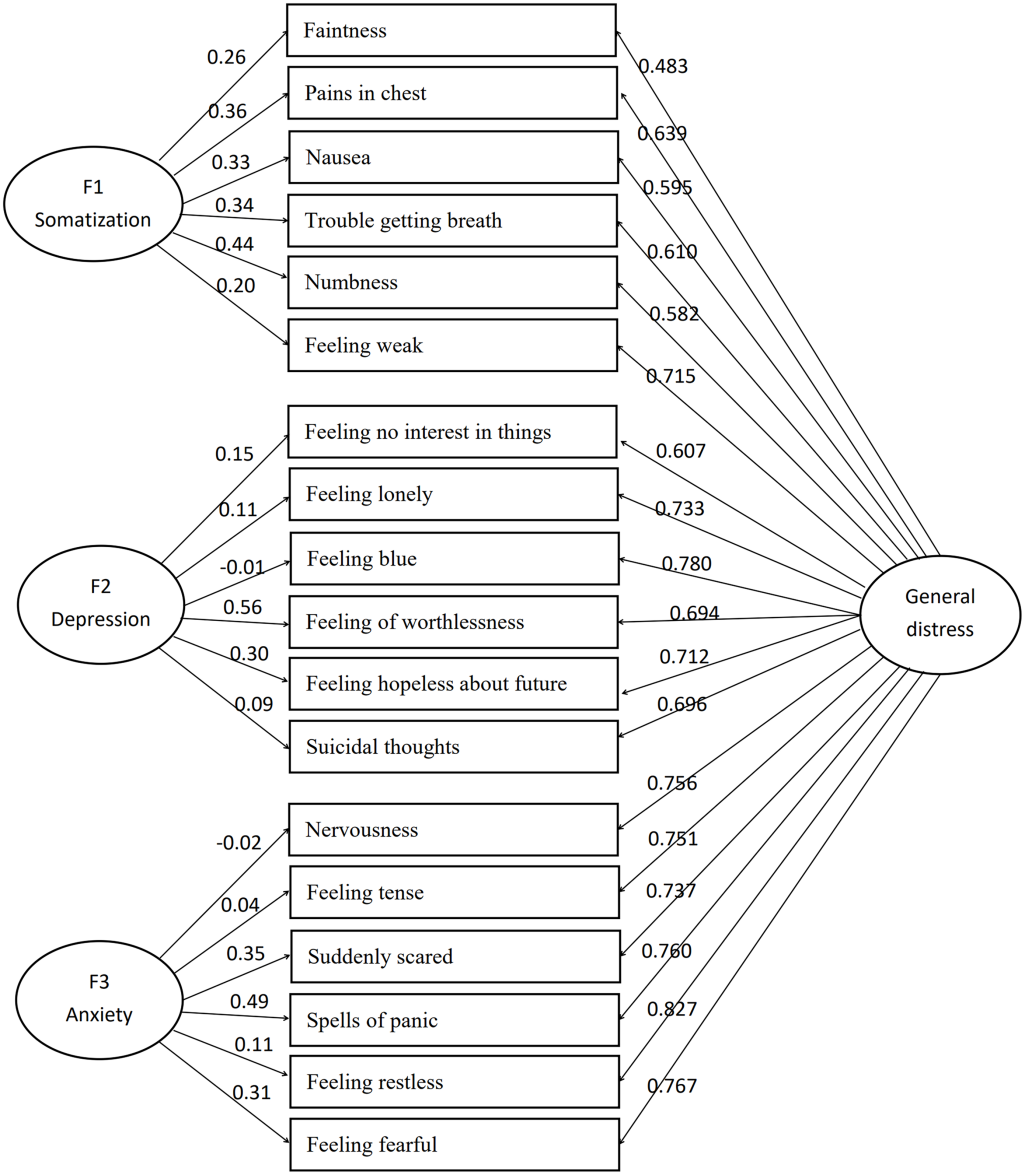
Structural model of three-factor bi-factor model.

The three-factor bi-factor model’s standardized factor loadings are shown in Table 4, and its structural model diagram in Figure 1.

**Table 4:** Standardized factor loadings for the BSI-18 bi-factor model.

Items		Somatization	Depression	Anxiety	General
Item 1	Faintness	0.260^***^			0.483^***^
Item 4	Pains in chest	0.357^***^			0.639^***^
Item 7	Nausea	0.326^***^			0.595^***^
Item 10	Trouble getting breath	0.343^***^			0.610^***^
Item 13	Numbness	0.436^***^			0.582^***^
Item 16	Feeling weak	0.200^***^			0.715^***^
Item 2	Feeling no interest in things		0.146^**^		0.607^***^
Item 5	Feeling lonely		0.107^*^		0.733^***^
Item 8	Feeling blue		−0.013		0.780^***^
Item 11	Feeling of worthlessness		0.564^***^		0.694^***^
Item 14	Feeling hopeless about future		0.300^***^		0.712^***^
Item 17	Suicidal thoughts		0.088^*^		0.696^***^
Item 3	Nervousness			−0.023	0.756^***^
Item 6	Feeling tense			0.043	0.751^***^
Item 9	Suddenly scared			0.345^***^	0.737^***^
Item 12	Spells of panic			0.489^***^	0.760^***^
Item 15	Feeling restless			0.110^**^	0.827^***^
Item 18	Feeling fearful			0.310^***^	0.767^***^

* *p* < 0.05;

** *p* < 0.01;

*** *p* < 0.001.

In terms of the reliability of this three-factor bi-factor model, the ω_H_ of the general factor was 0.918, and the ω_HS_ of three dimensions was 0.020 (somatization), 0.009 (depression), and 0.008 (anxiety). Meanwhile, the ECV was 85.7%.

### Measurement Invariance

As shown in Table 5, this study tested the model fits of the measurement invariance of the BSI-18 based on gender, living area, and educational level. The measurement invariance, which included configural, metric, scalar, and strict invariance, was tested sequentially between men and women. Although the TLI was close to 0.900 in the configural invariance, the remaining indices met the recommended requirements. Comparing the metric invariance model with the configural invariance model, results showed that △CFI had not changed and BIC was induced by 33.558. Comparing the scalar invariance model with the metric invariance model, △CFI = 0.004 and BIC was induced by 86.675. Comparing the strict invariance model with the scalar invariance model, △CFI = 0.003 and BIC was induced by 40.928. In all model comparisons, △CFIs were less than 0.010 and BIC values were decreased. Measurement equivalence was proven in both gender groups.

**Table 5:** Measurement invariance of the BSI-18.

Model	S-Bχ^2^	*df*	*p*	CFI	TLI	SRMR	RMSEA (90% CI)	BIC	△CFI	△BIC
**Gender**										
Configural invariance	824.680^*^	264	<0.001	0.909	0.894	0.046	0.048(0.044,0.052)	47667.797		
Metric invariance	838.907^*^	280	<0.001	0.909	0.900	0.058	0.047(0.043,0.050)	47634.239	0.000	33.558
Scalar invariance	873.458^*^	294	<0.001	0.905	0.902	0.058	0.046(0.043,0.050)	47547.564	0.004	86.675
Strict invariance	875.962^*^	312	<0.001	0.908	0.910	0.059	0.044(0.041,0.048)	47506.636	−0.003	40.928
**Living areas**										
Configural invariance	813.883^*^	264	<0.001	0.907	0.892	0.047	0.048(0.044,0.052)	46961.622		
Metric invariance	821.510^*^	280	<0.001	0.908	0.900	0.051	0.046(0.043,0.050)	46890.598	−0.001	71.024
Scalar invariance	855.256^*^	294	<0.001	0.905	0.901	0.051	0.046(0.042,0.049)	46802.875	0.003	87.723
Strict invariance	888.490^*^	312	<0.001	0.902	0.904	0.054	0.045(0.042,0.049)	46851.766	0.003	−48.891
**Educational levels**										
Configural invariance	806.387^*^	264	<0.001	0.911	0.897	0.045	0.047(0.044,0.051)	46968.945		
Metric invariance	814.976^*^	280	<0.001	0.912	0.904	0.051	0.046(0.042,0.049)	46895.609	−0.001	73.336
Scalar invariance	848.319^*^	294	<0.001	0.909	0.905	0.052	0.045(0.042,0.049)	46807.761	0.003	87.848
Strict invariance	897.440^*^	312	<0.001	0.904	0.906	0.056	0.045(0.042,0.049)	46890.413	0.005	−82.652

*χ*^2^, chi-square; df, degree of freedom; CFI, comparative fit index; TLI, Tucker-Lewis index; SRMR, standardized root-mean-square residual; RMSEA, root-mean-square error of approximation; BIC, Bayesian information criterion; and △*, change in the parameter.* *p < 0.05.

The measurement invariance between different living areas—rural and urban—was tested. Despite the TLI being close to 0.900 in the configural invariance, the rest of the indices met the recommendations. Comparing the metric invariance model with the configural invariance model, results showed that △CFI = 0.001 and BIC was induced by 71.024. Comparing the scalar invariance model with the metric invariance model, △CFI = 0.003 and BIC were induced by 87.723. Comparing the strict invariance model with the scalar invariance model, △CFI = 0.003 and BIC was increased by 48.891. The other indicators were acceptable. Overall, there is partial invariance in the strict invariance model.

Finally, the measurement invariance between groups with different educational levels was assessed. One group had received a higher college education and the other had not. While the TLI in the configural invariance was close to 0.900, all the remaining indices met the recommended requirements. Comparing the metric invariance model with the configural invariance model, results showed that △CFI = 0.001 and BIC were induced by 73.336. Comparing the scalar invariance model with the metric invariance model, △CFI = 0.003 and BIC was induced by 87.848. Comparing the strict invariance model with the scalar invariance model, △CFI = 0.005, BIC was increased by 82.652. The other indicators were acceptable. There was partial invariance in the strict invariance model.

## Discussion

In this study, the BSI-18 was used for the first time in adults with different backgrounds in China. Because of its convenience and efficiency, the BSI-18 is one of the most effective evaluation tools for screening individuals’ psychological symptoms. The results confirmed that the BSI-18 is equivalent in across gender, living area, and educational level variables.

The model comparison results show that the three-factor bi-factor model is the most concise, best-fitting, and most suitable factor structure model for the current data. The final model consists of one general distress factor and three specific dimensions, namely somatization, depression, and anxiety. The items loaded onto these same dimensions of the original theoretical model designed by Derogatis. The three-factor bi-factor model is consistent with research findings which used a sample of Chinese insurance industry participants (Li et al., 2018). These three types of psychological distress are both prevalent and relevant in the general Chinese adult population. According to previous studies, patients with severe depression are more likely to experience symptoms of anxiety (Fava et al., 2004; Kessler et al., 2008). Meanwhile, anxiety and depressive symptoms are closely related to the severity of physical symptoms, demonstrating high correlation and comorbidity with many chronic diseases (Huijbregts et al., 2010). The findings of this study offer significant extensions to existing research findings. The three-factor bi-factor model includes a global factor as well as the three special factors of somatization, depression, and anxiety. The global factor largely reflects the clinical comorbidity of somatization, anxiety, and depression symptoms. This study shows that the three dimensions and the general distress factor have a good reliability in Chinese adults.

In the final factor structure model of this study, loadings of several items were lower. Because the largest common loadings was explained by general factor, and the values of three factors at this point are the real indexes except the value of general factor. As a result, the loadings were lower. The study in Chinese insurance employees showed similar trends in factor loadings as our study. The loadings of item2, item5, item8 on depression were lower than the other items. On anxiety dimension, the item6 had weaker loadings. However, item3 also showed a lower loadings in our study. This is common that patterns of factor loadings differ from those expected based on the correlated factors model in some bifactor applications. Even so, factor loadings in some applications inconsistently change signs (some from positive to negative while others remain positive). Such results are not rare. Eid et al. (2017) found anomalous results in at least 50 (61%) of applications in their review of 82 bi-factor studies across different areas of psychology. Similar problems frequently occur in applications of the bi-factor approach to research on depression (Heinrich et al., 2021).

Measurement invariance is a requirement for any tool to ensure it will assess true differences across groups (Schmitt and Kuljanin, 2008). The current study shows that the BSI-18 exhibits measurement invariance across male and female, urban and rural, and high and low educated Chinese adults. The multi-group CFA results revealed that measurement invariance was confirmed in two of the three categories. Configural invariance suggests that the BSI-18’s three-factor bi-factor model structure is appropriate for adults of different genders, living in different areas, and with different levels of education. The factor structure of the BSI-18 was the same in each of these groups, both in terms of the general factor as well as the three specific factors (i.e., somatization, depression, and anxiety). Following the addition of the restriction conditions, the metric invariance was confirmed, indicating that the BSI-18 has the same measurement unit across different groups, and that when the latent factors changed by one unit, the observed variable also changed to the same degree. The findings revealed that the BSI-18 reached strict invariance across the different gender groups, and partial strict invariance across the different living areas and educational levels variables. These results indicate that cross-group comparisons are meaningful and that the BSI-18 can be used to compare differences in mental health between different groups. Therefore, it would be valuable to explore if females in fact experience more discomfort or whether they are simply more likely to admit the presence of distress in self-report studies. It is possible that males may sense a gendered constraint against acknowledging such feelings, as they may be perceived as weakness, even when completing in anonymous questionnaires. The current study is meaningful as it confirms that researchers can use the BSI-18 to compare the psychological symptoms of people with various levels of education to determine whether education has a positive or negative impact on mental health. Similarly, our findings indicate that this measure can but used to compare the mental health of those living across different regions, for example, to explore whether one’s living area or China’s hukou system has an impact on one’s mental health.

### Limitations and Future Research Directions

It is important to acknowledge that the present study also has some limitations. First, the study was based on a regional sampling of communities in Shaanxi Province. More research is needed to determine whether this conclusion applies to other Chinese regions. Second, the BSI-18 as a scale was originally developed for use in American samples, and the current study lacks a cross-cultural comparative test. Considering these limitations, future research should expand sampling areas both geographically and culturally in order to validate the results across other demographics.

## Conclusion

This study demonstrates that the BSI-18 is a convenient, reliable, and effective tool for screening for psychological symptoms, which can be used to screen general psychological distress in Chinese adults. The three-factor bi-factor model better reflects the BSI-18 factor structure in the adult Chinese population. Our findings showed that the BSI-18 has measurement invariance across adults from a variety of different backgrounds meaning that the BSI-18 scores can be used to reflect actual differences in psychological symptoms in Chinese adults.

## Data Availability Statement

The raw data supporting the conclusions of this article will be made available by the authors, without undue reservation.

## Ethics Statement

The studies involving human participants were reviewed and approved by the ethics committee of Xi’an Jiaotong University. The patients/participants provided their written informed consent to participate in this study. Written informed consent was obtained from the individual(s) for the publication of any potentially identifiable images or data included in this article.

## Author Contributions

YG and YW conceived and designed the study and contributed to data analysis supervision. YG, XN, YW, YQ, JF, and XL collected and input the data. YG wrote the manuscript. XN, YW, YQ, JF, and XL revised the manuscript. All authors contributed to the article and approved the submitted version.

## Funding

This research was funded by the National Social Science Fund of China (grant number: 19BSH121).

## Conflict of Interest

The authors declare that the research was conducted in the absence of any commercial or financial relationships that could be construed as a potential conflict of interest.

## Publisher’s Note

All claims expressed in this article are solely those of the authors and do not necessarily represent those of their affiliated organizations, or those of the publisher, the editors and the reviewers. Any product that may be evaluated in this article, or claim that may be made by its manufacturer, is not guaranteed or endorsed by the publisher.
